# Effect of Peanut Shell Extract and Luteolin on Gut Microbiota and High-Fat Diet-Induced Sequelae of the Inflammatory Continuum in a Metabolic Syndrome-like Murine Model

**DOI:** 10.3390/nu17142290

**Published:** 2025-07-10

**Authors:** Hemalata Deshmukh, Roberto Mendóza, Julianna M. Santos, Sathish Sivaprakasam, Moamen M. Elmassry, Jonathan M. Miranda, Patrick Q. Pham, Zarek Driver, Matthew Bender, Jannette M. Dufour, Chwan-Li Shen

**Affiliations:** 1 Department of Pathology, Texas Tech University Health Sciences Center, Lubbock, TX 79430, USA; hemalata.deshmukh@ttu.edu (H.D.); robert.mendoza@ttuhsc.edu (R.M.); julianna.santos@ttuhsc.edu (J.M.S.); patrick.q.pham@ttu.edu (P.Q.P.); zarek.driver@ttuhsc.edu (Z.D.); matthew.bender@ttuhsc.edu (M.B.); 2 Department of Biological Sciences, Texas Tech University, Lubbock, TX 79409, USA; 3 Woody L. Hunt School of Dental Medicine, Texas Tech University Health Sciences Center, El Paso, TX 79905, USA; 4 Department of Cell Biology and Biochemistry, Texas Tech University Health Sciences Center, Lubbock, TX 79430, USA; sathish.sivaprakasam@ttuhsc.edu (S.S.); jonathan.m.miranda@ttuhsc.edu (J.M.M.); jannette.dufour@ttuhsc.edu (J.M.D.); 5 Department of Molecular Biology, Princeton University, Princeton, NJ 08544, USA; elmassry@princeton.edu; 6 Department of Medical Education, Texas Tech University Health Sciences Center, Lubbock, TX 79430, USA; 7 Center of Excellence for Integrative Health, Texas Tech University Health Sciences Center, Lubbock, TX 79430, USA; 8 Obesity Research Institute, Texas Tech University, Lubbock, TX 79430, USA; 9 Center of Excellence for Translational Neuroscience and Therapeutics, Texas Tech University Health Sciences Center, Lubbock, TX 79430, USA

**Keywords:** bioactive compounds, luteolin, mitochondria, microbiome, colon, kidney, ileum, tissues, diabetes, mice

## Abstract

**Background:** Metabolic syndrome (MetS) is characterized by chronic inflammation, oxidative stress, and mitochondrial dysfunction. MetS is associated with increased intestinal permeability and dysbiosis. The objective of this study was to investigate the effects of peanut shell extract (PSE) and luteolin (LUT) on the kidneys, colon, and ileum in a MetS-like murine model. **Methods:** Thirty-six male Slc6a14^y/−^ mice were divided into four groups: low-fat diet (LFD), high-fat diet (HFD), HFD + 200 mg PSE/kg BW (PSE, p.o.), and HFD + 100 mg LUT/kg BW (LUT, p.o.) for 4 months. Outcome measures included glucose homeostasis, intestinal permeability, gut microbiome composition, and mRNA gene expression of mitochondrial homeostasis and inflammation/oxidative stress in the kidneys, colon, and ileum. **Results:** HFD resulted in glucose dysregulation with hyperglycemia and insulin resistance. PSE and LUT improved insulin tolerance and beta-cell function. PSE and LUT mitigated HFD-increased serum lipopolysaccharide-binding protein concentration. Perturbations in the gut microbiome were associated with HFD, and PSE or LUT reversed some of these changes. Specifically, *Phocaeicola vulgatus* was depleted by HFD and reverted by PSE or LUT. Relative to the LFD group, the HFD group (1) upregulated mitochondrial fusion (MFN1, MFN2, OPA1), mitophagy (TLR4, PINK1, LC3B), and inflammation (NFκB, TNFα, IL6), and (2) downregulated mitochondrial fission (FIS1, DRP1), biosynthesis (PGC1α, NRF1, NRF2, TFAM), electron transport chain (complex I), and antioxidant enzyme (SOD1) in the kidneys, colon, and ileum. **Conclusions:** PSE and LUT reversed such HFD-induced changes in the aforementioned gene expression levels.

## 1. Introduction

The growing incidence of diseases within the inflammatory continuum, such as metabolic syndrome (MetS), often concomitant type 2 diabetes mellitus (T2DM), and their chronic associated sequelae, is a major public health concern with global reach [1]. The World Health Organization estimates that 60% of all global deaths are attributable to chronic inflammatory diseases, including MetS, diabetes mellitus, obesity, cancer, cardiovascular disease, and autoimmune disease [2]. It is estimated that a quarter of all adults worldwide are affected by MetS, with approximately 30–40% of adults in the USA being affected [3].

Metabolic syndrome is characterized by a collection of derangements, including insulin resistance, hypertension, dyslipidemia, and central obesity, which together elevate the risk of conditions like cardiovascular disease and T2DM [4]. Emerging studies have highlighted gut dysbiosis as playing an important role in MetS and glucose homeostasis, influencing insulin sensitivity, inflammation, and the development of related diseases like T2DM [5,6,7]. Gut dysbiosis is closely associated with chronic inflammation and metabolic disturbances of the normal gut microenvironment, including (1) reduction in symbionts, (2) overgrowth of harmful microbes, and (3) loss of microbial diversity itself [8,9]. As a major site of microbial activity with the highest concentration of gut microbiota (≈10^12^ cells/mL), the colon is particularly vulnerable to such disturbances, especially considering factors such as antibiotics, poor diet, chronic stress, or illness [10]. Two other tissues, the ileum and kidneys, are also particularly exposed to these inflammatory signals relative to other organs, with the former playing a central role in immune sensing and nutrient absorption allowing for possibly greater antigen translocation, and the latter filtering circulating endotoxins and uremic toxins, activating proximal tubule epithelial cells to secrete cytokines and chemokines, where subsequent perpetuation of inflammatory cascades ensue [11,12].

Mitochondrial dysfunction, which acts as a potent inducer of inflammatory signaling in the gastrointestinal (GI) tissues, is a hallmark of MetS [13,14]. Such dysfunction can result in impaired adenosine triphosphate (ATP) production, accumulation of reactive oxygen species (ROS), activation of the NLRP3 inflammasome, and disruption of effector signaling pathways, contributing to the development of MetS [15,16]. Dysfunction of mitochondria can also compromise the integrity of the intestinal epithelial lining, particularly at tight junctions, leading to increased permeability and subsequent antigenemia-induced inflammation [17]. Furthermore, these aberrances have been implicated in damage to the renal and GI tissues, such as the colon and ileum, and disease progression in the context of MetS [16,17,18]. As the prevalence of MetS continues to rise, exploring novel therapeutic approaches is warranted to mitigate the impact of MetS on disrupted glucose homeostasis, gut dysbiosis, and mitochondrial dysfunction in GI tissues and overall health.

Peanut shell extract (PSE) is derived from the outer hull of peanuts and is rich in polyphenolic compounds which combat free radicals like ROS, leading to a reduction in general inflammation. Emerging evidence has substantiated the potent anti-inflammatory and antioxidant properties of PSE for its therapeutic role in obesity-induced diabetic animals [19,20]. Luteolin (LUT), a flavonoid antioxidant commonly found in fruits and vegetables [21] as well as in peanut shells [22], has been reported to mitigate inflammation-related diseases in animals with MetS [23,24,25] and diabetes [26,27] through its anti-inflammatory properties. Previous studies have demonstrated that PSE and LUT can lower blood glucose levels in diabetic mice, suggesting their potential in managing MetS [26,28,29]. For example, LUT reduces glycemic levels in diabetic mice [26], induces adipocyte browning [26], and ameliorates insulin resistance [27]. However, no studies that we know of have yet evaluated the effects of PSE or PSE-extracted LUT on the kidney-GI-microbiome axis using a MetS-like murine model.

In this study, we selected the kidney and two GI organs (colon and ileum) because all three tissue types are notably affected by microbial activity. Microbial activity within the colon allows for production of useful metabolites, namely short-chain fatty acids (e.g., acetate, propionate, and butyrate), which serve as critical energy substrates and modulators of host immune responses [30]. Disruption of short-chain fatty acid production contributes to systemic inflammation and metabolic dysfunction [10]. The kidneys, through their roles in metabolic homeostasis and filtration, and the ileum, as a key site of nutrient absorption and immune surveillance, particularly vulnerable to the systemic inflammation and oxidative stress associated with MetS [11,31]. Gut dysbiosis-associated alterations have been implicated in MetS, contributing to the production of harmful metabolites that potentially exacerbate MetS-affected organ damage [32].

We used Slc6a14^y/−^ gene knockout mice (analogous to the human SLC6A14 gene), which leads to disruption of the normal function of the solute carrier protein product that is involved in the transport of amino acids and other metabolites crucial for energy homeostasis. As a result, this genetic predisposition allows for a MetS-like state to develop, such as obesity, insulin resistance, dyslipidemia, and hypertension, where these gene knockout mice are prone to MetS symptomatology and associated T2DM development when the mice are placed on a HFD [33]. The chronic inflammation, oxidative stress, and mitochondrial dysfunction that characterize MetS are also well represented in Slc6a14^y/−^ mice, making them a robust MetS-like model for evaluating potential therapeutic interventions, such as PSE and PSE-extracted LUT for eventual human benefit [33].

This study aims to investigate the effects of PSE and PSE-extracted LUT on glucose homeostasis and GI health (in terms of gut microbiome composition and mitochondrial homeostasis) in mice with a predisposition toward a MetS-like state. We hypothesized that PSE and PSE-extracted LUT administration would increase insulin tolerance and sensitivity, decrease intestinal permeability and dysbiosis, and improve mitochondrial homeostasis and dynamics of renal and GI tissues in MetS-like mice. By examining gene expression profiles of mitochondrial homeostasis and markers of proinflammatory cytokines and antioxidant enzyme in the kidneys, colon, and ileum, we uncovered the potential therapeutic benefits of PSE and LUT in improving renal and GI function of MetS-like mice. Understanding these biochemical and molecular mechanisms can pave the way for the development of novel adjuvant interventions to mitigate the progression of MetS.

## 2. Materials and Methods

### 2.1. Animals and Treatments

A total of thirty-six male Slc6a14^y/−^ mice were utilized to investigate diet-induced obesity, hepatic steatosis, and a metabolic syndrome-like state, based on the model established in a previous study [33]. The mice were kindly provided by Dr. Vadivel Ganapathy, TTUHSC, Lubbock, TX, USA, and were housed under standard laboratory conditions. Mice were randomly divided into four experimental groups (n = 9 per group): (1) a low-fat diet (LFD) negative control group receiving diet D12450J (Research Diets, New Brunswick, NJ, USA); (2) a high-fat diet (HFD) positive control group fed diet D12492 containing 60% of calories from fat mainly from lard (Research Diets); (3) a treatment group receiving HFD along with PSE administered orally at 200 mg/kg BW per day; and (4) a second treatment group given HFD with LUT at a dose of 100 mg/kg BW per day by oral gavage. The intervention lasted for 4 months. PSE and LUT were administered at the aforementioned daily doses for 4 months. These doses were selected based on prior preclinical studies demonstrating beneficial effects on metabolic dysfunction and inflammation in high-fat diet-fed rodents [21,34,35,36]. The selected doses fall within the safe and effective range for chronic administration in murine models. Both the PSE (final extract ratio: 100:1 to 110:1, standardized to contain 20% LUT, Catalog number 1503) and the LUT (extracted from PSE with 99.04% LUT purity based on the results of high-performance liquid chromatography) were gifts provided by Sabinsa Corporation (East Windsor, NJ, USA).

Animals were housed two per cage under controlled environmental conditions (temperature: 22 ± 2 °C; relative humidity: 55 ± 5%) with a light/dark cycle of 12 h. Food and water were available ad libitum throughout the study duration. Weekly measurements included body weight, dietary intake, water consumption, and fasting blood glucose levels.

Based on our prior statistical power calculations [33], a sample size of 6–8 mice per group was sufficient to detect significant differences in insulin levels at α = 0.05 with 90% power. Thus, a group size of nine animals was selected to ensure adequate statistical robustness. All experimental procedures were approved by the Institutional Animal Care and Use Committee (IACUC) on 27 May 2022.

### 2.2. Sample Collection

Following the 4-month dietary intervention, mice were fasted for 4 h to assess blood glucose levels using a glucometer (Accu-Chek Aviva Glucose Meter, Roche Diabetes Care, Inc., Indianapolis, IN, USA). Animals were then anesthetized with isoflurane, and blood was collected via cardiac puncture. The mice were subsequently euthanized, and tissue samples—including kidneys, colon, ileum, and cecal feces—were harvested and immediately stored at −80 °C for future analyses [33]. Blood samples were centrifuged to separate the serum, which was also preserved at −80 °C for biochemical evaluations. Pancreatic tissue was fixed in Z-fix solution (AnaTech Ltd., Battle Creek, MI, USA) at room temperature for subsequent histological analysis of insulin and glucagon expression.

### 2.3. Glucose and Insulin Tolerance Tests

At baseline, and following the 4-month intervention period, all mice underwent glucose tolerance tests (GTT) and insulin tolerance tests (ITT) after a 4 h fasting period. For the GTT, glucose was administered intraperitoneally at a dose of 2 g/kg BW. For the ITT, insulin (Humulin; Abbott, Chicago, IL, USA) was delivered intraperitoneally at 1 U/kg BW. Blood glucose levels were measured from the tail vein at 0, 15, 30, 60, and 120 min post-injection using a glucometer (AmiStrip Plus, Germaine Laboratories, Inc., San Antonio, TX, USA) [20]. The area under the curve (AUC) for glucose levels during both tests was determined using the trapezoidal rule:

$$AUC\approx \sum_{i=1}^{n}\frac{\left(t_{i}-t_{i-1}\right)}{2}\left[G_{i}+G_{i-1}\right]$$

where t_i_ and t_i−1_ are time points, and G_i_ and G_i−1_ are corresponding glucose concentrations.

### 2.4. Serum Insulin and HOMA Assessment

Serum insulin concentrations were measured using a commercially available ELISA kit (EMD Millipore Co., Billerica, MA, USA). To evaluate insulin resistance, the HOMA-IR (Homeostatic Model Assessment for Insulin Resistance) index was calculated using the formula: HOMA-IR = [fasting glucose (mmol/L) × fasting insulin (pmol/L)] ÷ 22.5, where 22.5 is the normalization constant [20]. β-cell function was assessed by computing the HOMA-B (Homeostatic Model Assessment for Beta-Cell Function) index using the formula: HOMA-B = [20 × fasting insulin (pmol/L)] ÷ [fasting glucose (mmol/L) − 2.5], where 2.5 is the empirically derived physiological baseline for fasting glucose [20].

### 2.5. Histology on Pancreas Tissue

Pancreatic tissue sections were subjected to immunohistochemical staining to detect insulin and glucagon using guinea pig anti-insulin (1:1000 dilution; Dako Agilent Pathology Solutions, Santa Clara, CA, USA) and mouse anti-glucagon (1:5000 dilution; Millipore Sigma, Burlington, MA, USA) primary antibodies, respectively, following protocols described in our previous work [20]. Hematoxylin was used as a counterstain.

### 2.6. Measurement of Intestinal Permeability

Intestinal permeability was assessed by quantifying serum levels of lipopolysaccharide-binding protein (LBP) using a commercially available ELISA kit (LifeSpan Biosciences, Inc., Shirley, MA, USA).

### 2.7. Gene Profiling of Gut Microbiota and Data Analysis

Fecal microbial DNA was extracted using the PowerFecal DNA Isolation Kit (Qiagen Inc., Germantown, MD, USA). Sequencing of the V4 region of the 16S rRNA gene was carried out by MR DNA (Molecular Research LP, Shallowater, TX, USA), following protocols established in our previous publication [37]. Amplicons were purified with Ampure XP beads and subsequently used to construct DNA libraries for Illumina sequencing. The raw sequence data have been deposited in the NCBI BioProject database under accession number PRJNA1258714.

### 2.8. RNA Extraction and qRT-PCR

Total RNA was extracted from renal, colonic, and ileal tissues following procedures described in our previously published study [38]. Complementary DNA (cDNA) was synthesized from the isolated RNA and used for quantitative PCR amplification of target genes using specific primers listed in Supplement Table S1, in accordance with our earlier methodology [37]. Gene expression levels were normalized to β-actin as a housekeeping gene and calculated using the formula: x = 2^−(ΔCT×1000)^ as described by [39].

### 2.9. Statistical Analysis

All data are presented as mean ± standard error of the mean (SEM). Group comparisons were performed using one-way analysis of variance (ANOVA), followed by the Tukey post hoc test and the uncorrected Fisher least significant difference (LSD) test using version 9 of GraphPad Prism (GraphPad Software, San Diego, CA, USA). Statistical significance was denoted as follows: * *p* < 0.05, ** *p* < 0.005, *** *p* < 0.0005, **** *p* < 0.00005, and # for trends where 0.05 < *p* < 0.1.

For the gut microbiota analysis, sequencing depth median was 796,231 (1st quartile: 720,060—3rd quartile: 842,909) and amplicon sequence variants (ASVs) frequency median was 458,985 (1st quartile: 406,360—3rd quartile: 524,197) after quality filtering and denoising. Finally, we discovered the number of ASVs (7013). For taxonomy classification of ASVs, we used the Greengenes2 database. QIIME2 v. 2023.7 was used in 16S rRNA amplicon data analysis. For relative abundance statistical significance testing, we used LOCOM analysis (followed by Benjamini–Hochberg Procedure P_adj_ ≤ 0.05).

## 3. Results

### 3.1. PSE and LUT Improved Insulin Tolerance and Pancreatic Islet Function

**Figure 1:**
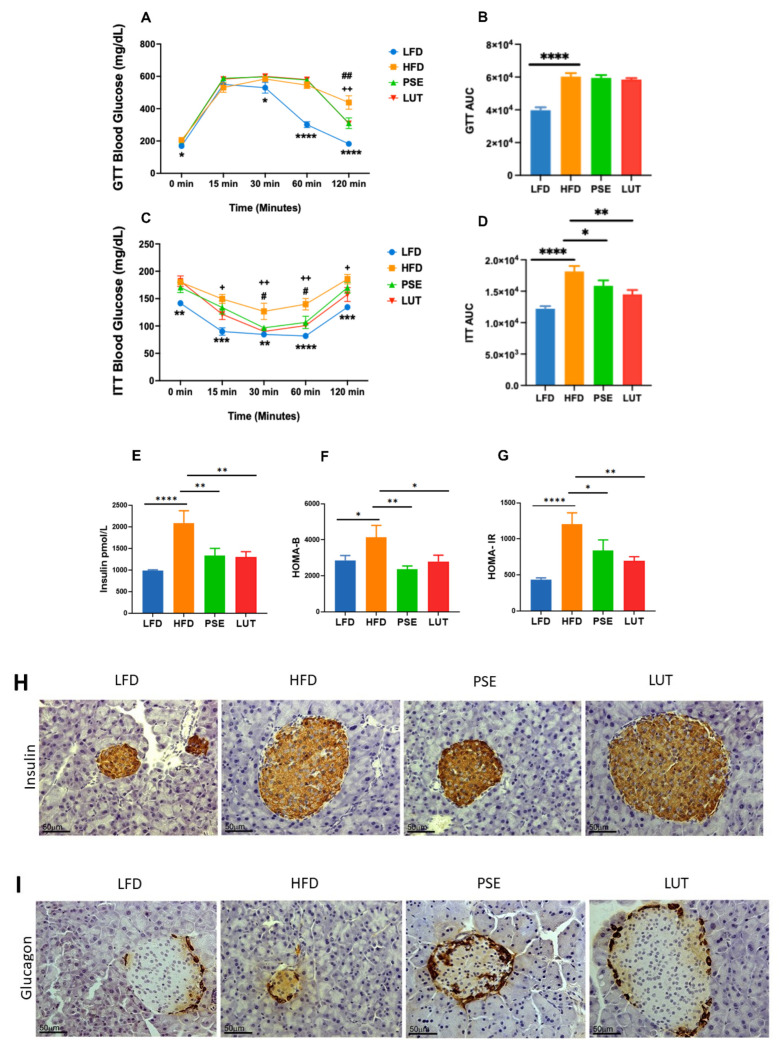
Effect of PSE and LUT on glucose homeostasis and pancreatic islet function: GTT (**A**), GTT AUC (**B**), ITT (**C**), ITT AUC (**D**), serum insulin (**E**), HOMA-IR (**F**), HOMA-B (**G**). n = 6–8 per group. The data are expressed as mean ± SEM and analyzed by one-way ANOVA followed by the Fisher LSD test. * HFD vs. LFD, # HFD vs. PSE, + HFD vs. LUT, * *p* < 0.05, ** *p* < 0.005, *** *p* < 0.0005, **** *p* < 0.00005. # *p* < 0.05, ## *p* < 0.005, + *p* < 0.05, ++ *p* < 0.005. Pancreatic tissue sections were immunostained for insulin ((**H**); marker of islet beta-cells) or glucagon ((**I**); marker of islet alpha-cells). Sections were counterstained with hematoxylin and images were 40× magnification.

The effects of PSE and LUT supplementation on glucose homeostasis (Figure 1) were assessed by GTT, ITT, serum insulin, HOMA-IR, HOMA-B, pancreatic insulin, and pancreatic glucagon. At the baseline, there were no differences in GTT, ITT, and respective GTT AUC and ITT AUC among the groups. At the end of the study, relative to the LFD group, the HFD group exhibited hyperglycemia (Figure 1A) and glucose intolerance (Figure 1B). Supplementation of PSE and LUT did not improve glucose intolerance in the obese Slc6a14^y/−^ mice, as shown by GTT AUC (Figure 1B).

Compared to the LFD group, the HFD group developed insulin resistance, as shown by increased glucose concentration (Figure 1C) along with AUC (Figure 1D) from ITT, as well as increased serum insulin levels (Figure 1E) and HOMA-IR (Figure 1F), indicative of diabetes. Supplementation of PSE and LUT to obese Slc6a14^y/−^ mice significantly improved insulin tolerance as demonstrated by the lower levels of ITT AUC, serum insulin, and HOMA-IR, compared with those without PSE and LUT (Figure 1D–F). PSE and LUT supplementation also significantly reduced HFD-induced HOMA-B relative to that in the LFD group (Figure 1G). Immunohistochemical staining of pancreatic tissue sections for insulin (Figure 1H) and glucagon (Figure 1I) revealed normal pancreatic islets with typical distribution of islet beta- and alpha-cells regardless of the treatment group.

### 3.2. PSE and LUT Decreased Intestinal Permeability

**Figure 2:**
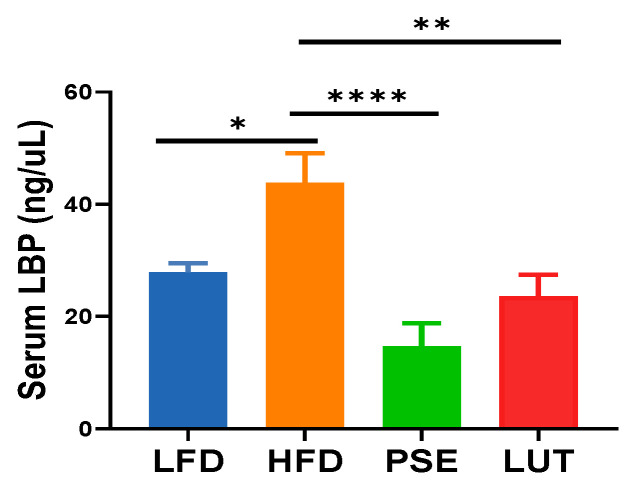
Effect of PSE and LUT on intestinal permeability in obese Slc6a14^y/−^ mice. Data are expressed as mean ± SEM, n = 7–9 per group, and analyzed by one-way ANOVA followed by the Tukey test. * *p* < 0.05, ** *p* < 0.005, **** *p* < 0.00005.

At the end of the study, we noted an increase in intestinal permability, as shown by increased LBP concentration in the plasma of the HFD group when compared to the LFD group. PSE and LUT supplementation significantly decreased plasma LBP concentration, suggesting decreased intestinal permeability (Figure 2).

### 3.3. Gut Microbiome Analysis

#### 3.3.1. Microbiome Alpha Diversity

**Figure 3:**
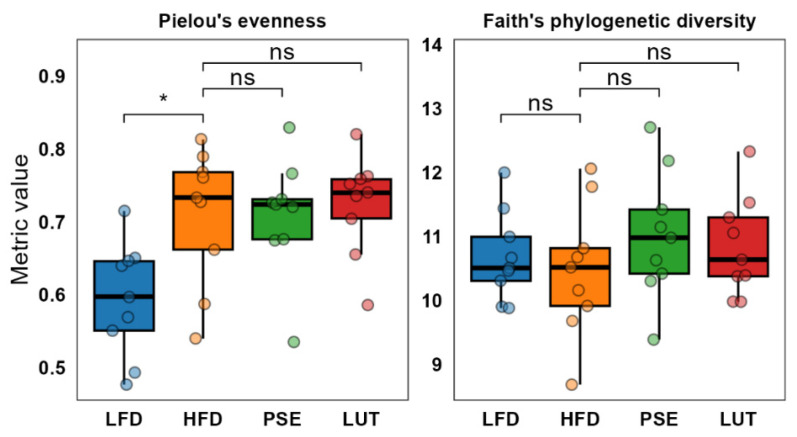
Alpha evenness and diversity metrics across different experimental groups. Kruskal–Wallis test followed by the Dunn test was used to determine statistical significance. * *p* < 0.05.

We first examined the microbiome alpha diversity between groups. The HFD group increased species evenness compared to the LFD group (*p* ≤ 0.05, Kruskal–Wallis test followed by the Dunn test on Pielou evenness). Supplementation with PSE or LUT did not change the HFD-induced effect in animals (*p* > 0.05). Unlike evenness, species richness across all groups did not change (*p* > 0.05, Kruskal–Wallis test followed by the Dunn test on Faith phylogenetic diversity) (Figure 3).

#### 3.3.2. Microbiome Beta Diversity

**Figure 4:**
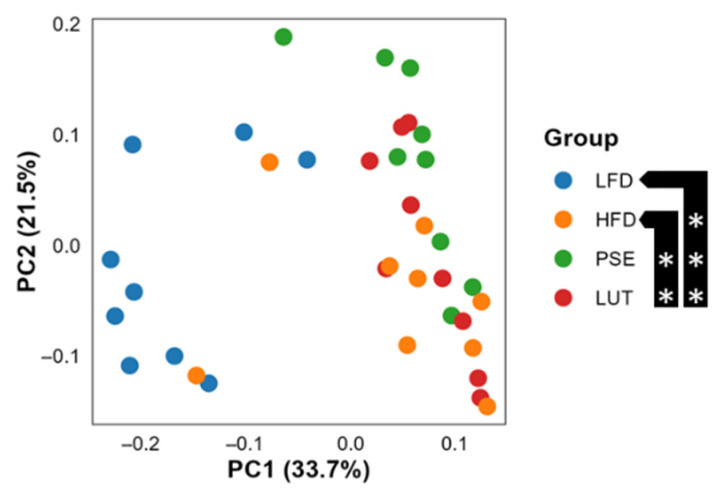
Beta diversity analysis of the gut microbiome across groups. PERMANOVA followed by pairwise comparison was used to determine statistical significance. ** *p* < 0.005, *** *p* < 0.0005.

We observed differences between the microbiome groups as indicated from weighted UniFrac distance measure (*p* ≤ 0.05, PERMANOVA followed by pairwise comparison) (Figure 4). Specifically, LFD vs. HFD, PSE, or LUT exhibited a statistically significant difference (*p* ≤ 0.05), as well as HFD vs. PSE or LUT (*p* ≤ 0.05). This indicates that the microbiome composition of all groups is potentially different, which might be reflected on the relative abundance of specific taxa.

#### 3.3.3. Microbiome Composition Analysis

**Figure 5:**
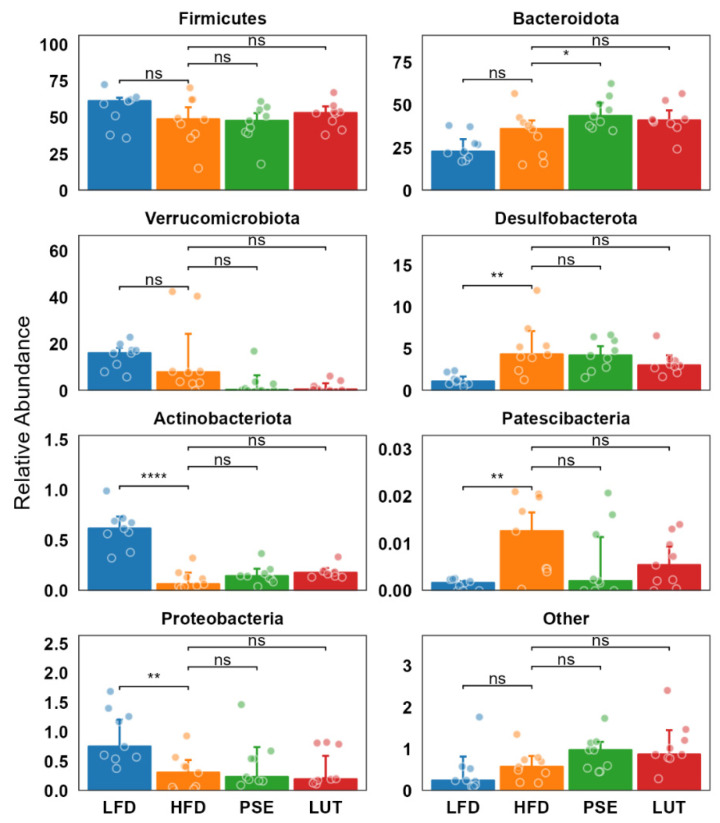
Effect of PSE and LUT on composition of the gut microbiome at the phylum level. * *p* < 0.05, ** *p* < 0.005, **** *p* < 0.00005.

We first examined the effects of PSE and LUT on microbiome composition in cecal feces (Figure 5). The most dominant phyla in all groups were Firmicutes, followed by Bacteroidota, comprising more than 85% of the community. Relative to the LFD group, the HFD group exhibited increased relative abundance of Desulfobacterota and Patescibacteria and decreased relative abundance of Actinobacteriota and Proteobacteria. Compared to the HFD group, only the PSE group, not the LUT group, had significantly increased abundance of the Bacteroidota phylum.

**Figure 6:**
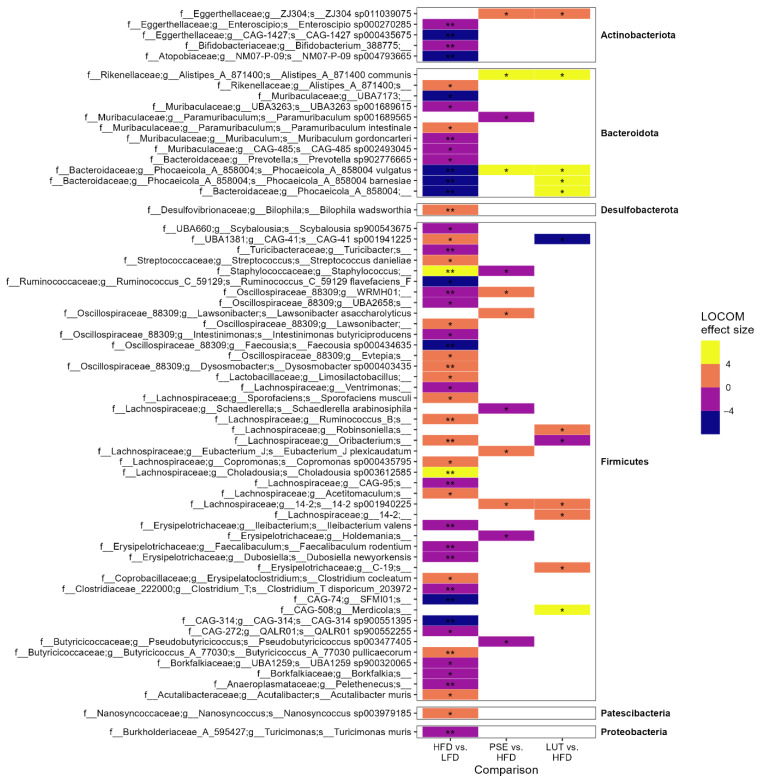
Effect of PSE and LUT on composition of the gut microbiome at ASVs level. LOCOM analysis was used to determine statistical significance, followed by FDR correction. * *p* < 0.05, ** *p* < 0.005.

Because we observed changes at this higher taxon level, we focused on the ASVs as they can be more informative. We observed changes in the relative abundance of ASVs in most phyla between HFD and LFD (Figure 6), including more than 50 ASVs that either increased or decreased in HFD. For instance, relative to the LFD group, the HFD group had greater abundance of two ASVs (*Staphylococcus* and *Choladousia* sp003612585), and lower abundance of nine ASVs (f_Eggerthellaceae, f_Muribaculaceae, f_Bacteroidaceae, f_Ruminococcaceae, f_Oscillospiraceae, f_CAG-74, and f_CAG-314). The addition of PSE or LUT reversed a few of the changes altered by HFD. Specifically, *Phocaeicola vulgatus* (previously known as *Bacteroides vulgatus*) was completely depleted by HFD (P_adj_ ≤ 0.01), yet after PSE or LUT supplementation, the species recovered and increased in abundance (P_adj_ ≤ 0.05).

**Figure 7:**
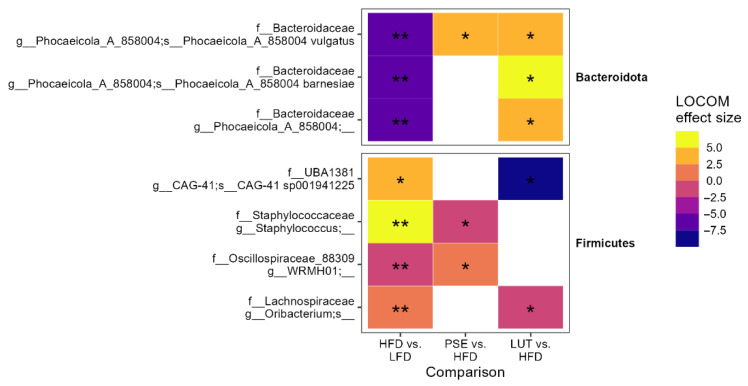
Gut microbiome ASVs reversed by PSE or LUT. LOCOM analysis was used to determine statistical significance, followed by FDR correction. Data in this figure are presented in Figure 6. * *p* < 0.05, ** *p* < 0.005.

Lastly, we focused on the changes in ASV abundance that were reverted by PSE or LUT supplementation (Figure 7). Among Bacteroidota phyla, both PSE and LUT supplementation significantly increased the abundance of HFD-depleted *Phocaeicola vulgatus*. LUT administration, not PSE, has shown to increase the abundance of two other HFD-depleted species (namely, *Phocaeicola barnesiae* and *Phocaeicola_A_858004*) in obese mice. On the other hand, PSE and LUT have differential effects on four species among *Firmicutes* phyla. For instance, relative to the HFD group, the PSE group had a decreased abundance of *Staphylococcus* and increased abundance of *WRMHO1*. Relative to the LFD group, LUT group showed decreased abundance of *CGA-41 sp001941225* and *Oribacterium*.

### 3.4. Mitochondrial Fusion Markers: MFN1, MFN2, OPA1

**Figure 8:**
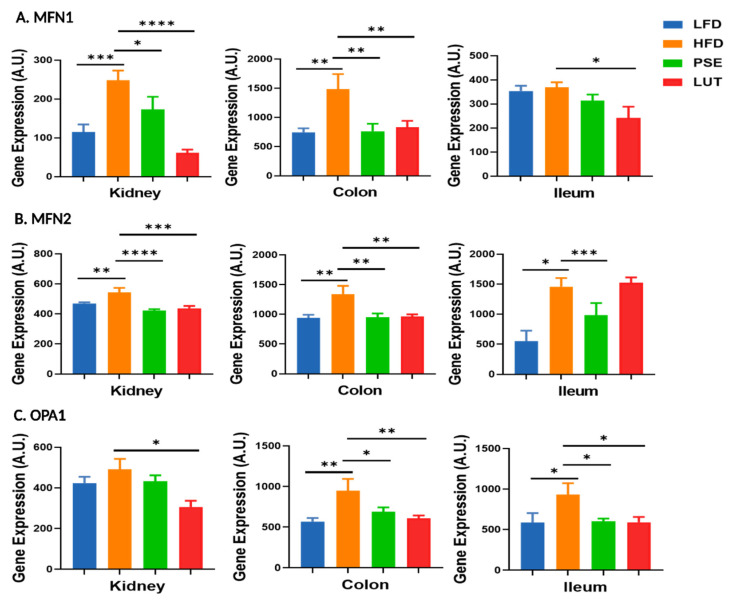
Effect of PSE and LUT on gene expression of MFN1 (**A**), MFN2 (**B**), and OPA1 (**C**) in the kidney, colon, and ileum. n = 6–8 per group. The data are expressed as mean ± SEM and analyzed by one-way ANOVA followed by the Fisher LSD test. * *p* < 0.05, ** *p* < 0.005, *** *p* < 0.0005, **** *p* < 0.00005.

Figure 8 shows the effects of PSE and LUT on mitochondrial fusion markers, namely MFN1 (Figure 8A), MFN2 (Figure 8B), and OPA1 (Figure 8C). The HFD mice had higher gene expression levels of MFN1 (kidney, colon), MFN2 (kidney, colon, and ileum) and OPA1 (colon and ileum) than those in the LFD mice. Administration of PSE and LUT to obese mice reverted such HFD-induced changes in MFN1, MFN2, and OPA1 gene expressions.

### 3.5. Mitochondrial Fission Markers: FIS1, DRP1

**Figure 9:**
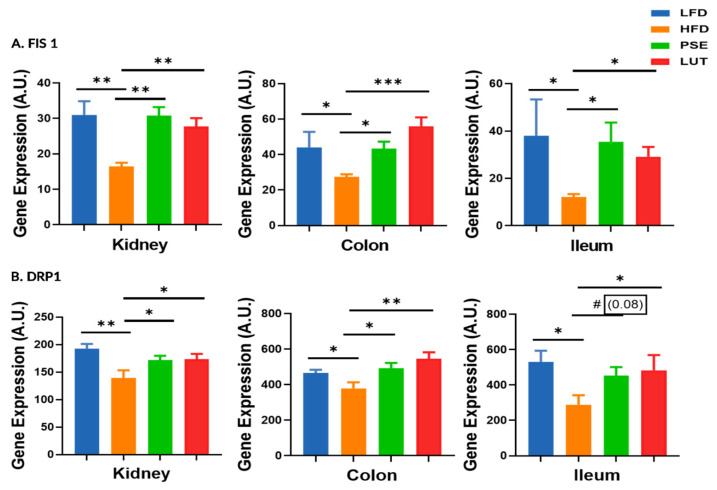
Effect of PSE and LUT on gene expression of FIS1 (**A**) and DRP1 (**B**) in the kidney, colon, and ileum. n = 6–8 per group. The data are expressed as mean ± SEM and analyzed by one-way ANOVA followed by the Fisher LSD test. * *p* < 0.05, ** *p* < 0.005, *** *p* < 0.0005, and # 0.05 < *p* < 0.1.

Compared to the LFD mice, the HFD mice had decreased FIS1 and DRP1 gene expression levels across the kidneys, colon, and ileum (Figure 9). Supplementation of PSE and LUT significantly restored the levels of HFD-induced FIS1 and DRP1 gene expression in these tissues.

### 3.6. Mitochondrial Biosynthesis-Associated Markers: PGC1α, NRF1, NRF2, TFAM

**Figure 10:**
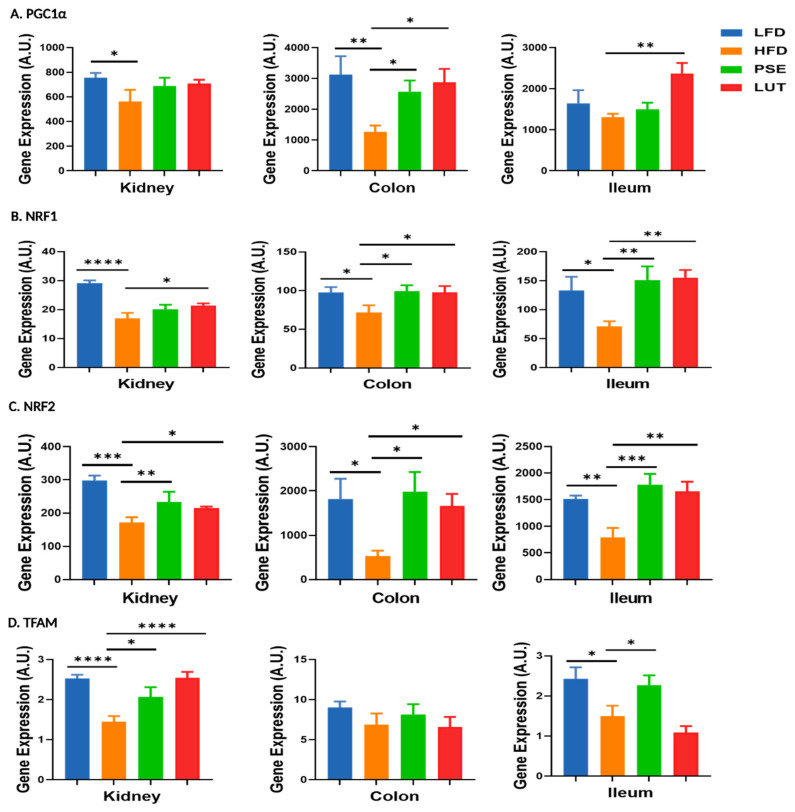
Effect of PSE and LUT on gene expression of PGC1α (**A**), NRF1 (**B**), NRF2 (**C**), and TFAM (**D**) in the kidney, colon, and ileum. n = 6–8 per group. The data are expressed as mean ± SEM and analyzed by one-way ANOVA followed by the Fisher LSD test. * *p* < 0.05, ** *p* < 0.005, *** *p* < 0.0005, and **** *p* < 0.00005.

We evaluated the impacts of PSE and LUT supplementation on the mitochondrial biosynthesis-associated markers, PGC1α (Figure 10A), NRF1 (Figure 10B), NRF2 (Figure 10C), and TFAM (Figure 10D) in the kidneys, colon, and ileum of mice. Relative to the LFD mice, the HFD mice had decreased mRNA expression levels of PGC1α (kidney, colon), NRF1 (all three tissues), NRF2 (all three tissues), and TFAM (kidney, ileum). PSE supplementation by oral gavage significantly restored these mRNA changes in the animals, as shown by increased gene expression levels of PGC1α (colon), NRF1 (colon, ileum), NRF2 (all three tissues), and TFAM (kidney, ileum). Similar to the PSE results, LUT administration also increased mRNA expression levels of PGC1α (colon, ileum), NRF1 (all three tissues), NRF2 (all three tissues), and TFAM (kidney).

### 3.7. Mitophagy-Associated Markers: TLR4, PINK1, LC3B

**Figure 11:**
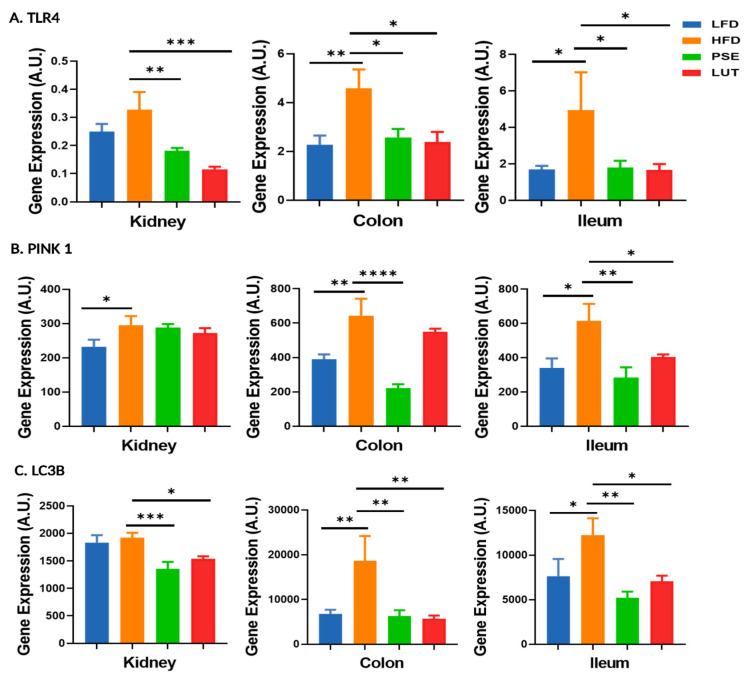
Effect of PSE and LUT on gene expression of TLR4 (**A**), PINK1 (**B**), and LC3B (**C**) in the kidney, colon, and ileum. n = 6–8 per group. The data are expressed as mean ± SEM and analyzed by one-way ANOVA followed by the Fisher LSD test. * *p* < 0.05, ** *p* < 0.005, *** *p* < 0.0005, and **** *p* < 0.00005.

Figure 11 shows the effects of PSE and LUT supplementation on mitophagy-associated markers, TLR4 (A), PINK1 (B), and LC3B (C) in kidney, colon, and ileum. In general, the HFD group had higher TLR4, PINK1, and LC3B gene expression levels in collected tissues, when compared to the LFD group. Relative to the HFD group, both PSE and LUT groups had lower TLR4, PINK1, and LC3B in all three tissues, except for kidney with no effects on PINK1 gene expression.

### 3.8. Mitochondrial Electron Transport Chain (ETC) Markers: Complex I, Complex III

**Figure 12:**
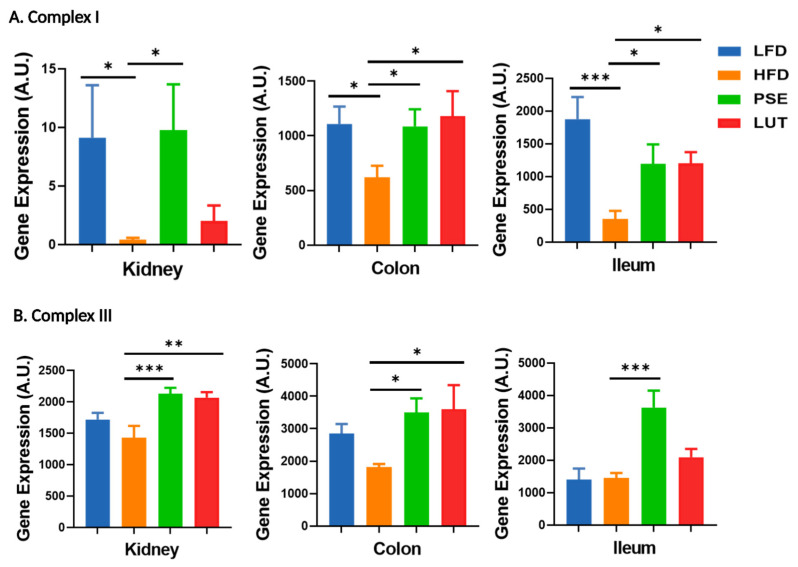
Effect of PSE and LUT on gene expression of complex I (**A**) and complex III (**B**) in the kidney, colon, and ileum. n = 6–8 per group. The data are expressed as mean ± SEM and analyzed by one-way ANOVA followed by the Fisher LSD test. * *p* < 0.05, ** *p* < 0.005, and *** *p* < 0.0005.

We assessed the effects of PSE and LUT on mitochondrial ETC markers complex I and complex III in the kidneys, colon, and ileum of obese mice (Figure 12). PSE and LUT administration increased both HFD-depleted complex I and complex III mRNA expression levels in these tissues.

### 3.9. Proinflammatory Cytokines and Antioxidant Enzyme: NFκB, TNFα, IL6, SOD1

**Figure 13:**
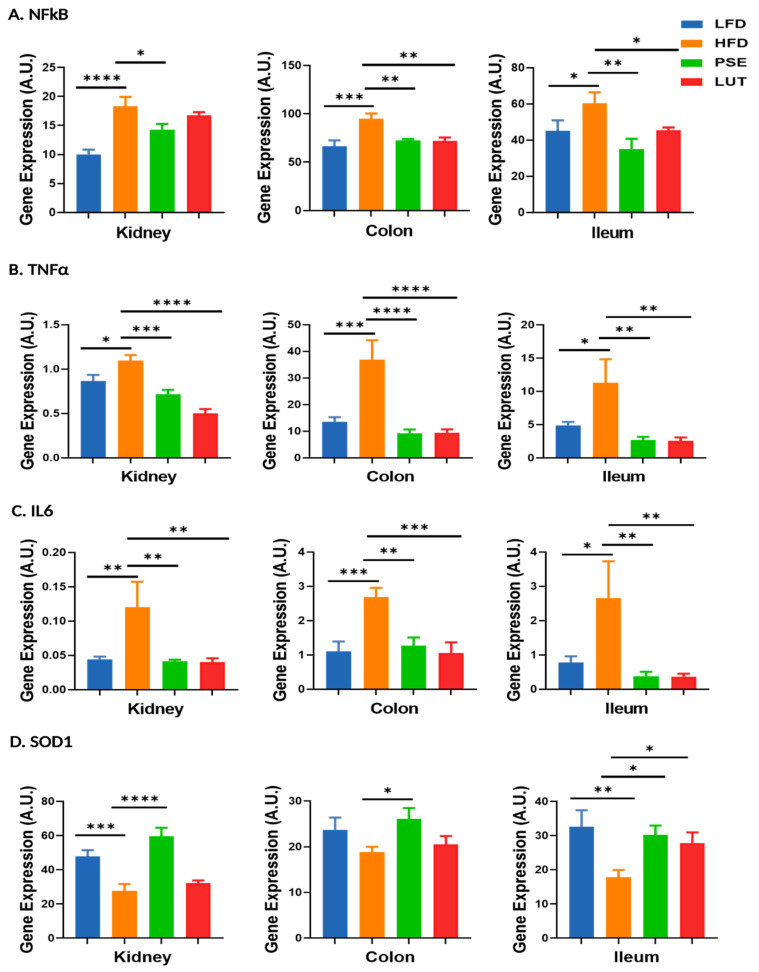
Effect of PSE and LUT on gene expression of NFκB (**A**), TNFα (**B**), IL6 (**C**), and SOD1 (**D**) in the kidney, colon, and ileum. n = 6–8 per group. The data are expressed as mean ± SEM and analyzed by one-way ANOVA followed by the Fisher LSD test. * *p* < 0.05, ** *p* < 0.005, *** *p* < 0.0005 and **** *p* < 0.00005.

We evaluated the impacts of PSE and LUT supplementation on the pro-inflammation cytokine markers [NFκB (Figure 13A), TNFα (Figure 13B) and IL6 (Figure 13C)] and antioxidant enzyme marker [SOD1 (Figure 13D)] in the kidneys, colon, and ileum of MetS-like mice. Compared to the LFD negative control mice, the HFD positive control mice had (1) increased gene expression levels of NFκB, TNFα, and IL6 in all three tissues and (2) decreased gene expression level of SOD1 in kidney and ileum of mice. PSE administration decreased HFD-induced change in gene expression levels for NFκB, TNFα, IL6, and increased SOD1 in all studied tissues. LUT administration had (1) inhibitory effects on all NFκB, TNFα, and IL6 gene expression levels, except for NFκB in the kidneys, and (2) stimulatory effects on SOD1 gene expression levels in ileum only.

## 4. Discussion

### 4.1. Glucose Homeostasis and Islet Cell Function in the Slc6a14^y/−^ Mouse Model

Using the Slc6a14^y/−^ mouse model, we successfully examined the effects of PSE and PSE-extracted LUT on glucose homeostasis, gut microbiota, and mitochondrial homeostasis in renal and GI tissues, namely the colon and ileum. Hyperglycemia and insulin resistance are key components of MetS. HOMA has been used to evaluate the interaction between glucose and insulin dynamics. HOMA-IR index is a measure of systemic insulin resistance, given the strong positive correlation between elevated HOMA-IR and the risk of developing MetS [40]. HOMA-B is a measure of beta-cell function in the pancreas; thus, an elevated HOMA-B value indicates that beta-cells are working harder to compensate for insulin resistance [41]. In MetS, insulin resistance (elevated HOMA-IR) and beta-cell dysfunction (elevated HOMA-B) are often present together since the initial response of the body to insulin resistance is often a compensatory elevation in systemic insulin levels, resulting in initially elevated HOMA-B [42]. In this study, we reported that both PSE and PSE-extracted LUT improved glucose homeostasis (Figure 1A–D) and insulin sensitivity (Figure 1E–G) in the obese Slc6a14^y/−^ mice, as shown in decreased AUC after ITT administration, serum insulin, HOMA-IR, and HOMA-B. Our findings of improved glucose homeostasis and insulin sensitivity by PSE are consistent with published studies on diabetic animals, including HFD/STZ rats [20,43,44], STZ rats [45], and Goto–Kakizaki rats [46]. Furthermore, our finding that PSE-extracted LUT alleviated MetS-like-induced insulin resistance in obese Slc6a14^y/−^ mice is corroborated by published works testing LUT-enriched artichoke leaf [23] and LUT-enriched *Tecoma stans* (L.) Juss. Ex Kunth [47] in HFD-induced MetS mice.

In MetS, persistent hyperglycemia leads to beta-cell dysfunction in the islets of the pancreas, with eventual failure resulting from systemic insulin resistance. Insulin resistance and beta-cell dysfunction are inextricably linked and form a self-inducing cycle during the progression through the MetS inflammatory continuum [48]. Our pancreatic immunohistochemical results (Figure 1H,I) found normal islet structure with alpha- and beta-cells expressing glucagon and insulin, respectively. Normal islet histology after HFD treatment is consistent with the start of the development of insulin resistance as the beta-cells first appear normal but later have impaired function as measured by elevated insulin secretion and HOMA-B. Treatment with PSE and LUT did not alter the normal islet histology but did return serum insulin and beta-cell function (HOMA-B) to normal. Such findings that PSE or LUT had no effects on glucagon and insulin in islet histology were not consistent with the study by Sun et al. [43]. Sun et al. reported that PSE improved the cellular structural and pathological changes in pancreatic islets of the HFD/STZ rats [43].

### 4.2. Gut Microbiome, Intestinal Barrier Function, and Metabolic Syndrome

The gut microbiome has been strongly associated with obesity and related metabolic disease states, including MetS, though the mechanisms involved have remained unclear. A permeable intestinal epithelium (increased intestinal permeability or disrupted intestinal barrier) can allow bacteria and bacterial components like lipopolysaccharides from the gut to enter the bloodstream (i.e., endotoxemia), triggering chronic low-grade inflammation, potential insulin resistance, and obesity, all of which are key factors in MetS progression [49]. The present study is the first to demonstrate the direct impact of PSE and LUT on reducing intestinal permeability in MetS-like mice with insulin resistance, as shown by decreased plasma LBP (an intestinal permeability marker, Figure 2). Such a finding agrees with the study by Sun et al. that LUT supplementation restores the damaged intestinal mucosal barrier and reduces intestinal permeability in rats with non-alcoholic fatty liver disease via the gut–liver axis [50].

The gut microbiome, a complex ecosystem of trillions of microorganisms residing predominantly in the colon, has emerged as having a crucial role in regulating metabolic inflammatory processes, notably in the pathogenesis of MetS within its inflammatory continuum [7,51]. Gut dysbiosis is an imbalanced gut microbiota composition that can disrupt the epithelial barrier, leading to increased intestinal permeability and chronic low-grade inflammation. This imbalance manifests itself through a shift in the microbial populations favoring pathogenic or opportunistic species over beneficial commensals, which facilitates the translocation of microbial-derived antigens such as lipopolysaccharides into systemic circulation [5,52]. Intriguingly, this study shows that PSE and LUT groups demonstrated a significant shift in gut microbiota beta diversity profiles compared to those in HFD groups (Figure 3), suggesting PSE and LUT administration significantly influenced microbiota composition of cecal feces in MetS-like mice. Such findings agree with published studies using LUT in a variety of disease models [53,54,55]. For instance, LUT has been shown to positively modulate gut microbiota beta diversity and mitigate gut microbiota dysbiosis in rodent models of inflammatory bowel disease [53], cadmium-induced liver and intestinal damage [54], and non-alcoholic fatty liver disease (now called metabolic dysfunction-associated steatotic liver disease or MASLD in humans) [55], potentially enhancing gut health and mitigating disease progression.

In general, mice and humans share two similar major phyla within their gut microbiota, namely Bacteroidetes and Firmicutes [56]. The development of MetS has been shown to be associated with decreased Bacteroidetes phylum, increased Firmicutes/Bacteroidetes ratio, and increased Proteobacteria phylum in the GI tract [57]. This study is the first study (Figure 7) to demonstrate both PSE and LUT administration exhibiting differential impacts on increasing the *Bacteroidota* genus (*P. vulgatus* in both PSE and LUT groups; *s. Phocaeicola_A_858005 barnesiae* in LUT group) in MetS-like mice. *P. vulgatus* has shown to be crucial for maintaining host health, including metabolic homeostasis, immunity, and gut–brain function [58]. The findings (Figure 7) that PSE and LUT supplementation reduced the abundance of *Firmicutes* genera or species (*f_Staphylococcaceae_g Staphylococcus* and *f_Oscillospiraceae_g_WRMHO1* in PSE group; *f_UBA1381_s_CAG-41 sp001941225* and *f_Lacnospiraceae_g_Oribacterium* in LUT group) agrees with the study by Gao et al., where it was reported that LUT attenuates *Staphylococcus aureus*-induced endometritis through inhibiting ferroptosis and inflammation via activating the NRF2 signaling pathway [59]. In addition, we also noted that the Firmicutes/Bacteroidetes ratio was also decreased in both PSE- and LUT-treated animals, providing evidence of their beneficial effects on the MetS-like state progression.

### 4.3. Mitochondrial Function, Biogenesis, and Inflammatory Modulation by PSE and LUT

Mitochondria are endosymbiotic cytoplasmic organelles that house the ETC on their inner mitochondrial membrane (IMM), which produces most adenosine triphosphate (ATP) molecules via oxidative phosphorylation, mediated by multiprotein complexes that shuttle electrons from nicotinamide adenine dinucleotide with hydrogen (NADH) and flavine adenine dinucleotide (FADH_2_) to molecular oxygen, the last electron acceptor in the series that ultimately forms physiologic water. This is achieved by the proton motive force (Δ*p*) including both a proton gradient (Δ*pH*) and an electron gradient known as the mitochondrial membrane potential (Δ*Ψmt*), where the Δ*pH* potential energy builds in the intermembrane space and the Δ*Ψmt* is shuttled down the IMM, to produce a synergistic Δ*p* at complex V (ATP synthase) for ATP gamma bond formation [33,60]. A compromised integrity or function of this structure and process increases uncontrolled ROS that serve as damaging catalysts for inflammatory cascades. It is important to note, however, ROS can serve as a signaling molecule in certain pathways when controlled [61]. When a single electron is transferred to molecular oxygen, it generates a highly reactive superoxide (O_2_^−•^), which, at physiological pH, is rapidly dismutated into hydrogen peroxide (H_2_O_2_) by superoxide dismutase—SOD1 in the cytoplasm and SOD2 in the mitochondrial matrix [60]. While there are at least 11 mitochondrial sites known to produce O_2_^−•^, complex I is the primary site in vivo, while complex III acts as an electron leak at the Qo site, especially as an underlying cause of non-communicable chronic disease, and is therefore part of the rationale behind this study [62].

As previously mentioned, mitochondrial dynamics are primarily a function of metabolic adaptation and quality control, with fusion increasing oxidative capacity and fission facilitating degradation of damaged organelles and their replacement [63]. Mitochondrial fusion is mediated through dynamin-related GTPases mitofusin 1 (MFN1) and optic atrophy type 1 (OPA1), which mediate outer and inner mitochondrial membrane fusion, respectively [18]. Meanwhile, mitochondrial fission is regulated by dynamin-related protein 1 (DRP1) through translocation to, and binding of, outer mitochondrial membrane (OMM) protein adaptors such as fission 1 (FIS1), which facilitate contact with the endoplasmic reticulum, so that constriction and isolation of the mitochondrion can ensue [64]. Such processes allow for an anomalous mitochondrion to be averaged out among the presumably more numerous healthy mitochondria so that, in the presence of a stressor, nuclear communication through the mitochondrial unfolded protein response can activate signaling pathways that induce transcription of nuclear genes associated with mitochondrial quality control and survival [65]. Alternatively, mitophagy of impaired mitochondria can be triggered through direct association pathways or via parkin-mediated polyubiquitination of OMM proteins, a process initiated by PTEN-induced kinase 1 (PINK1) [66,67]. In this context, toll-like receptor 4 (TLR4) plays a dual role: it not only contributes to innate immune signaling but also promotes mitochondrial quality control by initiating mitophagy under inflammatory stress [66]. One of the key downstream effectors is LC3B, a microtubule-associated protein that is lipidated and recruited to autophagosomal membranes to facilitate mitochondrial clearance [66,67]. Furthermore, biogenesis and maintenance of mitochondrial mass are coordinated primarily by PGC1α through the transcription of mtDNA and nuclear-encoded mitochondrial proteins [68]. This pathway also involves NRF2 and TFAM, which together contribute to mitochondrial DNA replication, transcription, and antioxidant defense [68]. First described as a thermogenic factor in adipose tissue, PGC1α was soon after shown to induce expression of ETC proteins, driving Δ*p* and mitochondrial dynamics [69]. Impairment of these dynamics, involving fission, fusion, mitophagy, and biogenesis, are crucial in the development of MetS. In the present study, both PSE and LUT supplementation decreased mitochondrial fusion markers (MFN1, MFN2, and OPA1), increased mitochondrial fission markers (FIS1 and DRP1), and decreased mitophagy markers (TLR4, PINK1, and LC3B) in the kidneys, colon, or ileum. Our current findings in the renal and GI tissues of MetS-like mice treated with PSE and LUT are consistent with previous studies in brain, liver, and adipose tissue of diabetic mice [19] and in brown fat development in in vitro 3T3-L1 adipocytes [26]. In addition, LUT has shown to reduce the initiation of excessive mitophagy in neuronal cell death via regulating autophagy and mitochondrial dynamics [70]. Such observations support our hypothesis that PSE and LUT improve mitochondrial dynamics in MetS-like mice.

Potential therapeutic means to mitigate mitochondrial dysfunction in MetS progression include enhancing mitochondrial biogenesis and ATP synthesis-associated enzymes [71]. In this study, both PSE and LUT administration have demonstrated (1) enhancing the mRNA levels of mitochondrial biogenesis markers (PGC1α, NRF1, NRF2, and TFAM) (Figure 10), (2) enhancing the mRNA levels of complex I and III (Figure 12), (3) enhancing the mRNA levels of antioxidant enzyme markers (SOD1) (Figure 13), and (4) decreasing the mRNA levels of inflammation markers (NFκB, TNFα, IL6) (Figure 13) in the kidneys, colon, and ileum. Our findings of mitochondrial biogenesis in the studied tissues are supported by previous studies in a variety of animal tissues [19,26]. For instance, PSE has been reported to increase the expression levels of genes like NRF1, NRF2, and TFAM in liver, brain, and white adipose tissue of db/db mice [19]. Liu et al. reported that both PSE and LUT treatments increased the gene expression levels of brown adipocyte-specific markers, like UCP1, PGC1α, and SIRT1, potentially contributing to mitochondrial biogenesis of brown tissues [26]. In this study, our findings that LUT mitigates inflammation markers in MetS-like mice corroborate previous studies that LUT (1) protects against diabetic cardiomyopathy by inhibiting NFκB-mediated inflammation and activating the NRF2-mediated antioxidant responses in STZ-induced diabetic mice [72] and (2) reverses MetS-induced biochemical dysfunction and related cardiac injury via the suppression of apoptosis, inflammation, and stress in rats with MetS-associated cardiac injury [24].

## 5. Conclusions

Chronic inflammation and oxidative stress are central contributors to insulin resistance, beta-cell dysfunction, and tissue injury in MetS development. PSE and LUT administration to MetS-like mice improved glucose homeostasis, insulin sensitivity, and mitochondrial function in the kidney, colon, and ileum organs. Recovery in mitochondrial integrity could be, at least in part, linked to restoration of intestinal barrier integrity and a shift in the gut microbiome.

While this study provides mechanistic insights into the role gut dysbiosis plays in mitochondrial dysfunction and subsequent inflammatory sequelae, several limitations must be acknowledged. First, the findings are based exclusively on preclinical data from Slc6a14^y/−^ male mice, limiting extrapolation to human physiology; moreover, future studies should either incorporate female cohorts with homozygous and/or heterozygous females to account for sex-specific or genotype-specific effects. Second, the oral doses of PSE (200 mg/kg of BW) and LUT (100 mg/kg of BW) used in this study do not yet reflect clinically equivalent or bioavailable concentrations for humans. Third, all MetS criteria were met in this murine model with the exception of direct measurement of lipid profiles for dyslipidemia, a characteristic feature of MetS, which is an important caveat and is why we term this model “MetS-like”; however, the model was presumed to exhibit dyslipidemia based on the prior study consistently demonstrating hepatic steatosis and increased NAS and fat accumulation scores in Slc6a14^y/−^ mice fed a HFD [33]. Lastly, it is unclear whether the observed anti-inflammatory and antioxidant effects of PSE and LUT are mediated by direct cellular actions or indirectly via bioactive metabolites produced through microbial biotransformation, particularly in the gut. This uncertainty is especially relevant for LUT, which is known to exhibit poor oral bioavailability due to rapid metabolism and limited absorption [73], though this factor supports the observed positive shift in microbial composition. This may necessitate the future development of enhanced delivery systems (e.g., nanoemulsions, liposomal formulations, or co-administrations with bio-enhancers) for human application [74].

In terms of future research, this work establishes a strong foundation for investigating PSE and LUT as gut–microbiome–mitochondria ameliorating agents in metabolic disease. Future clinical trials are warranted to validate the glucose-lowering, anti-inflammatory, and microbiota-modulating effects of PSE and/or LUT from animal findings to the individuals with MetS or sequalae within the inflammatory continuum.

## Data Availability

The raw sequencing data (BioProject access number PRJNA1258714) of 16S rRNA amplicon sequencing was deposited in the National Center for Biotechnology Information BioProject database. The original contributions presented in this study are included in the article. Further inquiries can be directed to the corresponding authors.

## Supplementary Materials

The following supporting information can be downloaded at: https://www.mdpi.com/article/10.3390/nu17142290/s1, Table S1: List of primers for mRNA.

## Abbreviations

BW: body weight; NLRP3: NOD-, LRR-, and pyrin domain-containing protein 3; PTEN: phosphatase and tensin homolog; UCP1: uncoupling protein 1; SIRT1: silent information regulator type 1; NAS: non-alcoholic fatty liver disease score.

## References

[1] WHO Global Report on Diabetes 2023. https://www.who.int/health-topics/diabetes#.

[2] Noubiap J.J., Nansseu J.R., Lontchi-Yimagou E., Nkeck J.R., Nyaga U.F., Ngouo A.T., Tounouga D.N., Tianyi F.-L., Foka A.J., Ndoadoumgue A.L. (2022). Geographic distribution of metabolic syndrome and its components in the general adult population: A meta-analysis of global data from 28 million individuals. Diabetes Res. Clin. Pract..

[3] Liang X., Or B., Tsoi M.F., Cheung C.L., Cheung B.M.Y. (2023). Prevalence of metabolic syndrome in the United States National Health and Nutrition Examination Survey 2011–18. Postgrad. Med. J..

[4] Hayden M.R. (2023). Overview and New Insights into the Metabolic Syndrome: Risk Factors and Emerging Variables in the Development of Type 2 Diabetes and Cerebrocardiovascular Disease. Medicine.

[5] Crudele L., Gadaleta R.M., Cariello M., Moschetta A. (2023). Gut microbiota in the pathogenesis and therapeutic approaches of diabetes. eBiomedicine.

[6] Chong L.L.T., Chong C.K., Jensen S.L., Lau K.M. (2024). Gut microbiota in type 2 diabetes mellitus: A systematic review of compositional and functional alterations. Front. Endocrinol..

[7] Craciun C.-I., Neag M.-A., Catinean A., Mitre A.-O., Rusu A., Bala C., Roman G., Buzoianu A.-D., Muntean D.-M., Craciun A.-E. (2022). The Relationships between Gut Microbiota and Diabetes Mellitus, and Treatments for Diabetes Mellitus. Biomedicines.

[8] Patloka O., Komprda T., Franke G. (2024). Review of the Relationships Between Human Gut Microbiome, Diet, and Obesity. Nutrients.

[9] Boicean A., Ichim C., Sasu S.M., Todor S.B. (2025). Key Insights into Gut Alterations in Metabolic Syndrome. J. Clin. Med..

[10] de Vos W.M., Tilg H., Van Hul M., Cani P.D. (2022). Gut microbiome and health: Mechanistic insights. Gut.

[11] Li X.J., Shan Q.Y., Wu X., Miao H., Zhao Y.Y. (2024). Gut microbiota regulates oxidative stress and inflammation: A double-edged sword in renal fibrosis. Cell Mol Life Sci..

[12] Daryabor G., Atashzar M.R., Kabelitz D., Meri S., Kalantar K. (2020). The Effects of Type 2 Diabetes Mellitus on Organ Metabolism and the Immune System. Front. Immunol..

[13] Masenga S.K., Kabwe L.S., Chakulya M., Kirabo A. (2023). Mechanisms of Oxidative Stress in Metabolic Syndrome. Int. J. Mol. Sci..

[14] Ezenabor E.H., Adeyemi A.A., Adeyemi O.S. (2024). Gut Microbiota and Metabolic Syndrome: Relationships and Mechanisms. Int. J. Endocrinol..

[15] Cojocaru K.-A.L.I., Goriuc A., Antoci L.-M., Ciobanu C.-G., Popescu R., Vlad C.-E., Blaj M., Foia L.G. (2023). Mitochondrial dysfunction, oxidative stress, and therapeutic strategies in diabetes, obesity, and cardiovascular disease. Antioxidants.

[16] Todosenko N., Khaziakhmatova O., Malashchenko V., Yurova K., Bograya M., Beletskaya M., Vulf M., Gazatova N., Litvinova L. (2023). Mitochondrial Dysfunction Associated with mtDNA in Metabolic Syndrome and Obesity. Int. J. Mol. Sci..

[17] Smith S.A., Ogawa S.A., Chau L., Whelan K.A., Hamilton K.E., Chen J., Tan L., Chen E.Z., Keilbaugh S., Fogt F. (2021). Mitochondrial dysfunction in inflammatory bowel disease alters intestinal epithelial metabolism of hepatic acylcarnitines. J. Clin. Investig..

[18] Kabootari M., Habibi Tirtashi R., Amouzegar A., Masoumi S., Azizi F., Amouzegar A. (2025). Changes in metabolic syndrome status and risk of chronic kidney disease over a decade of follow-up in the Iranian population. Sci. Rep..

[19] Deshmukh H., Santos J.M., Bender M., Dufour J.M., Lovett J., Shen C.L. (2024). Peanut Shell Extract Improves Mitochondrial Function in db/db Mice via Suppression of Oxidative Stress and Inflammation. Nutrients.

[20] Bender M., Santos J.M., Dufour J.M., Deshmukh H., Trasti S., Elmassry M.M., Shen C.-L. (2024). Peanut Shell Extract Improves Markers of Glucose Homeostasis in Diabetic Mice by Modulating Gut Dysbiosis and Suppressing Inflammatory Immune Response. Nutrients.

[21] Zhu M., Sun Y., Su Y., Guan W., Wang Y., Han J., Wang S., Yang B., Wang Q., Kuang H. (2024). Luteolin: A promising multifunctional natural flavonoid for human diseases. Phytother. Res..

[22] Peng M.M., Chen Z.S., Deng Q.P., Zhu S.J., Wang G. (2021). The roles of luteolin in peanut shell extract—Mediated protection of erythrocytes against hypoxanthine-xanthine oxidase-induced toxicity. Food Biosci..

[23] Kwon E.Y., Kim S.Y., Choi M.S. (2018). Luteolin-Enriched Artichoke Leaf Extract Alleviates the Metabolic Syndrome in Mice with High-Fat Diet-Induced Obesity. Nutrients.

[24] Dai X.Y., Liang B., Sun Y.L. (2025). Luteolin ameliorates rat model of metabolic syndrome-induced cardiac injury by apoptosis suppression and autophagy promotion via NR4A2/p53 regulation. BMC Complement. Med. Ther..

[25] Fikry H., Saleh L.A., Sadek D.R., Alkhalek H.A.A. (2025). The possible protective effect of luteolin on cardiovascular and hepatic changes in metabolic syndrome rat model. Cell Tissue Res..

[26] Liu W.R., Wang L.H., Zhang J. (2022). Peanut Shell Extract and Luteolin Regulate Lipid Metabolism and Induce Browning in 3T3-L1 Adipocytes. Foods.

[27] Xu N., Zhang L., Dong J., Zhang X., Chen Y.-G., Bao B., Liu J. (2014). Low-dose diet supplement of a natural flavonoid, luteolin, ameliorates diet-induced obesity and insulin resistance in mice. Mol. Nutr. Food Res..

[28] Kahksha, Alam O., Al-Keridis L.A., Khan J., Naaz S., Alam A., Ashraf S.A., Alshammari N., Adnan M., Beg A. (2023). Evaluation of Antidiabetic Effect of Luteolin in STZ Induced Diabetic Rats: Molecular Docking, Molecular Dynamics, In Vitro and In Vivo Studies. J. Funct. Biomater..

[29] Sangeetha R. (2019). Luteolin in the management of type 2 diabetes mellitus. Curr. Res. Nutr. Food Sci. J..

[30] Thaiss C.A., Zeevi D., Levy M., Zilberman-Schapira G., Suez J., Tengeler A.C., Abramson L., Katz M.N., Korem T., Zmora N. (2014). Transkingdom Control of Microbiota Diurnal Oscillations Promotes Metabolic Homeostasis. Cell.

[31] Li H., Zou L., Long Z., Zhan J. (2025). Immunometabolic alterations in type 2 diabetes mellitus revealed by single-cell RNA sequencing: Insights into subtypes and therapeutic targets. Front. Immunol..

[32] Wahlström A., Sayin S.I., Marschall H.U., Bäckhed F. (2016). Intestinal Crosstalk between Bile Acids and Microbiota and Its Impact on Host Metabolism. Cell Metab..

[33] Sivaprakasam S., Sikder M.O., Ramalingam L., Kaur G., Dufour J.M., Moustaid-Moussa N., Wachtel M.S., Ganapathy V. (2021). SLC6A14 deficiency is linked to obesity, fatty liver, and metabolic syndrome but only under conditions of a high-fat diet. BBA-Mol. Basis Dis..

[34] Taweesap P., Potue P., Khamseekaew J., Iampanichakul M., Jan-O B., Pakdeechote P., Maneesai P. (2025). Luteolin Relieves Metabolic Dysfunction-Associated Fatty Liver Disease Caused by a High-Fat Diet in Rats Through Modulating the AdipoR1/AMPK/PPARgamma Signaling Pathway. Int. J. Mol. Sci..

[35] Tan X., Yang Y., Xu J., Zhang P., Deng R., Mao Y., He J., Chen Y., Zhang Y., Ding J. (2019). Luteolin Exerts Neuroprotection via Modulation of the p62/Keap1/Nrf2 Pathway in Intracerebral Hemorrhage. Front. Pharmacol..

[36] Gao A.X., Xia T.C.-X., Peng Z.-T., Wu Q.-Y., Zhu Y., Dong T.T.-X., Tsim K.W.-K. (2023). The ethanolic extract of peanut shell attenuates the depressive-like behaviors of mice through modulation of inflammation and gut microbiota. Food Res. Int..

[37] Shen C.-L., Wang R., Santos J.M., Elmassry M.M., Stephens E., Kim N., Neugebauer V. (2024). Ginger alleviates mechanical hypersensitivity and anxio-depressive behavior in rats with diabetic neuropathy through beneficial actions on gut microbiome composition, mitochondria, and neuroimmune cells of colon and spinal cord. Nutr. Res..

[38] Santos J.M., Deshmukh H., Elmassry M.M., Yakhnitsa V., Ji G., Kiritoshi T., Presto P., Antenucci N., Liu X., Neugebauer V. (2024). Beneficial Effects of Ginger Root Extract on Pain Behaviors, Inflammation, and Mitochondrial Function in the Colon and Different Brain Regions of Male and Female Neuropathic Rats: A Gut-Brain Axis Study. Nutrients.

[39] Rao X., Huang X., Zhou Z., Lin X. (2013). An improvement of the 2^(-delta delta CT) method for quantitative real-time polymerase chain reaction data analysis. Biostat. Bioinforma Biomath..

[40] Yoon H., Jeong D.K., Lee K.S., Kim H.S., Moon A.E., Park J. (2016). Relationship between metabolic syndrome and metabolic syndrome score and beta cell function by gender in Korean populations with obesity. Endocr. J..

[41] Wallace T.M., Levy J.C., Matthews D.R. (2004). Use and abuse of HOMA modeling. Diabetes Care.

[42] Thomas D.D., Corkey B.E., Istfan N.W., Apovian C.M. (2019). Hyperinsulinemia: An Early Indicator of Metabolic Dysfunction. J. Endocr. Soc..

[43] Sun X.-M., Ye H.-Q., Liu J.-B., Wu L., Lin D.-B., Yu Y.-L., Gao F. (2018). Assessment of anti-diabetic activity of peanut shell polyphenol extracts. J. Zhejiang Univ. B.

[44] Shehnaz S.I., Roy A., Vijayaraghavan R., Sivanesan S. (2023). Luteolin Mitigates Diabetic Dyslipidemia in Rats by Modulating ACAT-2, PPARalpha, SREBP-2 Proteins, and Oxidative Stress. Appl. Biochem. Biotechnol..

[45] Xiao C., Chen M.Y., Han Y.P., Liu L.J., Yan J.L., Qian L.B. (2023). The protection of luteolin against diabetic cardiomyopathy in rats is related to reversing JNK-suppressed autophagy. Food Funct..

[46] Queiroz M., Leandro A., Azul L., Figueirinha A., Seiça R., Sena C.M. (2021). Luteolin Improves Perivascular Adipose Tissue Profile and Vascular Dysfunction in Goto-Kakizaki Rats. Int. J. Mol. Sci..

[47] Morales-Ferra D.L., Zavala-Sanchez M.A., Jimenez-Ferrer E., Gonzalez-Cortazar M., Zamilpa A. (2022). Effect of Tecoma stans (L.) Juss. ex Kunth in a Murine Model of Metabolic Syndrome. Plants.

[48] Hudish L.I., Reusch J.E.B., Sussel L. (2019). β Cell dysfunction during progression of metabolic syndrome to type 2 diabetes. J. Clin. Investig..

[49] Fändriks L. (2017). Roles of the gut in the metabolic syndrome: An overview. J. Intern. Med..

[50] Sun W.-L., Yang J.-W., Dou H.-Y., Li G.-Q., Li X.-Y., Shen L., Ji H.-F. (2021). Anti-inflammatory effect of luteolin is related to the changes in the gut microbiota and contributes to preventing the progression from simple steatosis to nonalcoholic steatohepatitis. Bioorg. Chem..

[51] Sinha A.K., Laursen M.F., Brinck J.E., Rybtke M.L., Hjørne A.P., Procházková N., Pedersen M., Roager H.M., Licht T.R. (2024). Dietary fibre directs microbial tryptophan metabolism via metabolic interactions in the gut microbiota. Nat. Microbiol..

[52] Wang P.X., Deng X.R., Zhang C.H., Yuan H.J. (2020). Gut microbiota and metabolic syndrome. Chin. Med. J..

[53] Yang S., Duan H., Yan Z., Xue C., Niu T., Cheng W., Zhang Y., Zhao X., Hu J., Zhang L. (2025). Luteolin Alleviates Ulcerative Colitis in Mice by Modulating Gut Microbiota and Plasma Metabolism. Nutrients.

[54] Zou H., Ali W., Deng K., Chen Y., Sun J., Wang T., Ma Y., Liu Z. (2024). The protective effect of luteolin on cadmium induced liver intestinal toxicity in chicken by Gut-liver axis regulation. Poult. Sci..

[55] Liu X., Sun R., Li Z., Xiao R., Lv P., Sun X., Olson M.A., Gong Y. (2021). Luteolin alleviates non-alcoholic fatty liver disease in rats via restoration of intestinal mucosal barrier damage and microbiota imbalance involving in gut-liver axis. Arch. Biochem. Biophys..

[56] Nguyen T.L.A., Vieira-Silva S., Liston A., Raes J. (2015). How informative is the mouse for human gut microbiota research?. Dis. Model. Mech..

[57] Festi D., Schiumerini R., Eusebi L.H., Marasco G., Taddia M., Colecchia A. (2014). Gut microbiota and metabolic syndrome. World J. Gastroenterol..

[58] Jin S., Chen P., Yang J., Li D., Liu X., Zhang Y., Xia Q., Li Y., Chen G., Li Y. (2024). alleviates diet-induced metabolic dysfunction-associated steatotic liver disease progression by downregulating histone acetylation level via 3-HPAA. Gut Microbes.

[59] Gao S.Y., Gao Y.J., Cai L.F., Qin R. (2024). Luteolin attenuates Staphylococcus aureus-induced endometritis through inhibiting ferroptosis and inflammation via activating the Nrf2/GPX4 signaling pathway. Microbiol. Spectr..

[60] Park J.W., Voss P.G., Grabski S., Wang J.L., Patterson R.J. (2008). Association of galectin-1 and galectin-3 with Gemin4 in complexes containing the SMN protein. Nucleic Acids Res..

[61] Rigoulet M., Yoboue E.D., Devin A. (2011). Mitochondrial ROS generation and its regulation: Mechanisms involved in H(2)O(2) signaling. Antioxid. Redox Signal..

[62] Wong H.S., Dighe P.A., Mezera V., Monternier P.A., Brand M.D. (2017). Production of superoxide and hydrogen peroxide from specific mitochondrial sites under different bioenergetic conditions. J. Biol. Chem..

[63] Chen W., Zhao H., Li Y. (2023). Mitochondrial dynamics in health and disease: Mechanisms and potential targets. Signal Transduct. Target. Ther..

[64] Losón O.C., Song Z.Y., Chen H.C., Chan D.C. (2013). Fis1, Mff, MiD49, and MiD51 mediate Drp1 recruitment in mitochondrial fission. Mol. Biol. Cell..

[65] Shpilka T., Haynes C.M. (2018). The mitochondrial UPR: Mechanisms, physiological functions and implications in ageing. Nat. Rev. Mol. Cell Biol..

[66] Nguyen T.N., Padman B.S., Lazarou M. (2016). Deciphering the Molecular Signals of PINK1/Parkin Mitophagy. Trends Cell Biol..

[67] Koyano F., Yamano K., Kosako H., Kimura Y., Kimura M., Fujiki Y., Tanaka K., Matsuda N. (2019). Parkin-mediated ubiquitylation redistributes MITOL/March5 from mitochondria to peroxisomes. Embo Rep..

[68] Scarpulla R.C., Vega R.B., Kelly D.P. (2012). Transcriptional integration of mitochondrial biogenesis. Trends Endocrinol. Metab..

[69] Puigserver P., Wu Z., Park C.W., Graves R., Wright M., Spiegelman B.M. (1998). A cold-inducible coactivator of nuclear receptors linked to adaptive thermogenesis. Cell.

[70] Vongthip W., Nilkhet S., Boonruang K., Sukprasansap M., Tencomnao T., Baek S.J. (2024). Neuroprotective mechanisms of luteolin in glutamate-induced oxidative stress and autophagy-mediated neuronal cell death. Sci. Rep..

[71] Prasun P. (2020). Mitochondrial dysfunction in metabolic syndrome. Biochim. Biophys. Acta Mol. Basis Dis..

[72] Li L., Luo W., Qian Y., Zhu W., Qian J., Li J., Jin Y., Xu X., Liang G. (2019). Luteolin protects against diabetic cardiomyopathy by inhibiting NF-κB-mediated inflammation and activating the Nrf2-mediated antioxidant responses. Phytomedicine.

[73] Dabeek W.M., Marra M.V. (2019). Dietary Quercetin and Kaempferol: Bioavailability and Potential Cardiovascular-Related Bioactivity in Humans. Nutrients.

[74] Ezike T.C., Okpala U.S., Onoja U.L., Nwike C.P., Ezeako E.C., Okpara O.J., Okoroafor C.C., Eze S.C., Kalu O.L., Odoh E.C. (2023). Advances in drug delivery systems, challenges and future directions. Heliyon.

