# Supplementary material for: Effect of Peanut Shell Extract and Luteolin on Gut Microbiota and High-Fat Diet-Induced Sequelae of the Inflammatory Continuum in a Metabolic Syndrome-like Murine Model
Source: Nutrients. 2025 Jul 10;17(14):2290. doi: 10.3390/nu17142290 (PMC12299383; doi:10.3390/nu17142290)
Supplement: Supplementary file 1 [file nutrients-17-02290-s001.zip › nutrients-3689431-supplementary.pdf]

Table S1. List of primers for mRNA.

Genes	Forward	Reverse
MFN1	5′-AGC TCG CTG TCA TTG GGG AG-3′	5′-TCC CTC CAC ACT CAG GAA GC-3′
MFN2	5′- TCC TGA ACA ACC GCT GGG AT-3′	5′-GAT CCA CCA CGC CTA GCT CA-3′
OPA1	5′- CAG CTG GCA GAA GAT CTC AAG -3′	5′- CAT GAG CAG GAT TTT GAC ACC -3′
Drp1	5′- ACA ACA GGA GAA GAA AAT GGA GTT G-3′	5′- AGA TGG ATT GGC TCA GGG CT-3′
FIS1	5′-CTG CGG TGC AGG ATG AAA GAC-3′	5′-GGC GTA TTC AAA CTG CGT GCT-3′
TFAM	5′ -GCT TCC AGG GGG CTA AGG ATG-3′	5′-TCG CCC AAC TTC AGC CAT TT-3′
PGC-1α	5′-CAG GAG CTG GAT GGC TTG GG-3′	5′-GGG CAA AGA GGC TGG TCC T-3′
NRF1	5′-AGC AGC CGT TGG AGC ACT TA-3′	5′-CGT CAC GGC TTT GCT GAT GG-3′
NRF2	5′- CTC TCT GGA GAC GGC CAT GAC T-3′	5′- CTG GGC TGG GGA CAG TGG TAG T-3′
TLR4	5′-TTG CAT CTG GCT GGG ACT CTG-3′	5′-TTC AGG GGG TTG AAG CTC AGA T-3′
PINK1	5′ -TCG GCC TGT CAG GAG ATC CA-3′	5′-CAT TGC AGC CCT TGC CGA TG-3′
LC3B	5′ -CAT GCC GTC CGA GAA GAC CT-3′	5′-CCG GAT GAG CCG GAC ATC TT-3′
Complex I (NDUFC1)	5′-GGT TTG TCT ACA TCG GCT TCC - 3′	5′-TAC AGA AGC TGG CGA TGC AAA -3′
Complex III (UQCRC1)	5′-GCA GTC CTC GCA TCC TAC CT -3′	5′-CTC CCG AGT GCT GTA GGC AT-3′
NFkB	5′-CCT CCA CCC CGA CGT ATT GC-3′	5′-GCC AAG GCC TGG TTT GAG AT-3′
TNF α	5′- GAA CTC CAG GCG GTG TCT GT-3′	5′ - CTG AGT GTG AGG GTC TGG GC-3′
IL6	5′-AGC CAC TGC CTT CCC TAC TTC-3′	5′ -GAC AGT GCA TCA TCG CTG TTC AT-3′
SOD1	5′-AGG GCG TCA TTC ACT TCG AG-3′	5′-ACA TGC CTC TCT TCA TCC GCT-3′
β-actin	5′-ACA ACC TTC TTG CAG CTC CTC C-3′	5′-TGA CCC ATA CCC ACC ATC ACA-3′
